# Frequency of Extravasation on Pericatheter Retrograde Urethrogram in Patients Who Undergo Posterior Urethroplasty

**DOI:** 10.7759/cureus.10041

**Published:** 2020-08-26

**Authors:** Sami Ullah, Sundas Karimi, Munir Ahmed, Farah Yasmin, Asfand Yar Cheema, Mohit Bhagia, Vijaya Chaitanya Bollampally, Ehtesham Zahoor, Abdul-Malik Kassim, Umar Farooque, Syed Adeel Hassan, Naresh Kumar

**Affiliations:** 1 Urology, Pakistan Navy Ship Shifa Hospital, Karachi, PAK; 2 General Surgery, Combined Military Hospital, Karachi, PAK; 3 Urology and Transplantation, Jinnah Post Graduate Medical Centre, Karachi, PAK; 4 Cardiology, Dow University of Health Sciences, Karachi, PAK; 5 Medicine, Lahore Medical & Dental College, Lahore, PAK; 6 Urology, B.K.L. Walawalkar Rural Medical College & Hospital, Kasarwadi, IND; 7 Neurological Surgery, Capital Medical University, Beijing, CHN; 8 Internal Medicine, Liaquat College of Medicine and Dentistry, Darul Sehat Hospital, Karachi, PAK; 9 Urology, American University of Antigua School of Medicine, Osbourn, ATG; 10 Neurology, Dow University of Health Sciences, Karachi, PAK; 11 Internal Medicine, Dow University of Health Sciences, Karachi, PAK; 12 Medicine, Dow University of Health Sciences, Karachi, PAK

**Keywords:** extravasation, posterior urethral stricture, retrograde pericatheter urethrogram, posterior urethroplasty, catheter removal

## Abstract

Introduction

Urethroplasty is the gold standard treatment for urethral stricture disease resulting from pelvic fractures, urethral manipulation, and straddle injuries. Post-operative morbidity depends on the presence of urethral catheterization with or without a suprapubic catheter (SPC). Urethral healing at the anastomotic site can be easily assessed using retrograde pericatheter urethrography (RPU). Post-operative removal of the catheter is traditionally performed on the 21^st^ day following urethroplasty. However, some controversy still exists regarding the best feasible time of proper urethral healing and its assessment utilizing simple techniques. The duration of anastomotic healing differs depending on the type of procedure performed, but whether there is any significant difference in duration of healing at the anastomotic site according to the etiology of short‐segment stricture urethra is still a dilemma.

Materials and methods

This was a descriptive case-series conducted for a duration of six months from September 2019 to February 2020 at the urology department of a tertiary care hospital in Karachi, Pakistan. A sample population of 135 patients aged 20-50 years with posterior urethral stricture who underwent posterior urethroplasty with disease duration of >12 months was included in the study. All patients were put on the next operation theater (OT) list for urethroplasty. After surgery, the patients were catheterized and were kept in the ward under observation for 48 hours and discharged on the 2^nd^ post-operative day. All patients were followed weekly and RPU was performed on the 21^st^ day following urethroplasty to assess the presence of extravasation and the collected data was entered into the proforma by the investigators. All statistical analysis was performed using Statistical Package for Social Sciences (SPSS) version 23.0.

Results

The mean age of our participants was 32.8±6.02 years. The mean duration of the procedure was 26.3±7.14 months. Extravasation cases were observed in less than one-fifth (n=22 out of 135, 16.3%) of the posterior urethral stricture patients in our study.

Conclusions

It is to be concluded that extravasation is fairly common in patients who undergo posterior urethroplasty. The prevalence varies depending on the assessment method, likely reflecting the treatment of somatic symptoms.

## Introduction

Male urethral stricture is defined as the narrowing and/or chronic fibrosis of the lumen of the male urethra. It is a highly prevalent disease accounting for nearly 5000 inpatient visits and half a million office visits annually in the United States [1]. The incidence of this disease is estimated to be 200-1,200 cases per 100,000 individuals with a marked rise in those aged above 55 years. Stricture can be further subdivided into two types, namely anterior and posterior, varying in location and underlying pathogenesis. In a retrospective analysis comprising urethral stricture cases presenting for reconstruction at a single institution, most of the strictures were anterior (92.2%) with nearly half of these occurring in the bulbar urethra (46.9%), followed by penile (30.5%), penile and bulbar (9.9%), and panurethral (4.9%) strictures. Stricture diseases profoundly affect the quality of life (QOL) of an individual, resulting in infections or severe cases that can lead to bladder calculi, fistulas, sepsis, and ultimately renal failure [2]. Urethral stricture management is complex and depends on the characteristic features of the stricture. Published literature suggests no difference between urethral dilation and internal urethrotomy with regards to long-term outcomes with a wide range of success rates between 8-80% and long-term success rates of 20-30% [3].

Urethroplasty is frequently performed for the stricture of the urethra. No current standard guidelines exist regarding the catheter removal following urethroplasty. Urinary catheters are usually removed approximately three to four weeks after urethroplasty; however, some studies demonstrate the safe removal of urinary catheters at even earlier intervals ranging from three to eight days depending on the technique [4, 5]. However, postoperative healing is affected by multiple preoperative and intraoperative variables and governs the shorter or longer duration of catheterization individual patient variability [6].

Anastomotic healing of urethroplasty is usually accessed by micturating cystourethrogram (MCU) requiring catheter removal. Peri-catheter retrograde urethrogram (pcRUG) is a safe technique that allows for adequate urethral assessment and healing following urethroplasty before the removal of the indwelling catheter. In the case of inadequate healing, the option to keep the catheter exists without risk of reinsertion trauma that is present in the case of the MCU. Extravasation of contrast from the urethra during this procedure is suggestive of incomplete healing and indicates the need for delayed removal of urinary catheters [7-9]. Varying incidence of extravasation on postoperative pcRUG in patients who undergo posterior urethroplasty is reported ranging from 11.5% to 14.8% [10, 11]. The usual time of catheter removal after urethroplasty varies from seven to 21 days, depending on urethral healing [2, 4, 5]. The primary objective of this study was to determine the frequency of extravasation on pcRUG as it is important to investigate the status of it so that the treatment of such patients can be anticipated in an appropriate clinical line and appropriate diagnosis is made to prevent postoperative complications. A secondary aim was to access the impact of socio-demographic and clinical factors on the presence of extravasation in our patients.

## Materials and methods

Study setting and design

This was a descriptive case-series carried out at the urology department of a tertiary care hospital in Karachi, Pakistan from September 2019 to February 2020 over a period of six months.

Sample size, inclusion and exclusion criteria

A sample size of 135 patients was calculated using the World Health Organization (WHO) sample size calculator by using a frequency of 14.8% [11] of extravasation on postoperative pcRUG in patients who undergo posterior urethroplasty with a 5% margin of error at a confidence interval of 95%. All patients aged 20-50 years with posterior urethral stricture who underwent posterior urethroplasty with the disease duration of >12 months were included in this study. We excluded all patients with enlarged prostate diagnosed on digital rectal examination and ultrasonography (weight more than 20 mg) and patients with no evidence of urethral stricture on urethrogram/urethroscopy. We also excluded all patients with bladder stones diagnosed on ultrasound pelvis and x-ray pelvis and those with neurogenic bladder diagnosed on history and urodynamic studies.

Sampling technique and data collection

A non-probability convenience-based sampling technique was employed to collect data. Pre-operatively, both written and verbal informed consent was taken from each patient by the primary investigator of this study. The purpose and benefits of the study along with necessary operation details were explained to all the participants. The preparation of the patient for surgery was done in a standard manner as being done for any urethroplasty. All patients were put on the next operation theater (OT) list for urethroplasty. All the surgical procedures were done by the researcher himself under the supervision of a consultant urologist with more than five years’ experience, to control surgeon bias. After surgery, the patients were catheterized and kept in the ward under observation for 48 hours. All patients were discharged on the 2nd postoperative day unless otherwise indicated soaked dressing. The principal investigator followed the patients weekly and on the 21st day following urethroplasty, pcRUG was performed to determine the presence of extravasation. Other variables such as age and duration of the procedure were also noted on the pre-defined proforma. An exclusion criterion was followed strictly to control cofounders and bias in the study results.

Statistical analysis

All statistical analysis was performed using Statistical Package for Social Sciences (SPSS) version 23.0. Continuous variables such as age and duration of the procedure were presented as mean and standard deviation whereas frequencies and percentages were calculated for the categorical outcome variable i.e. extravasation with dichotomous options (yes/no). Effect modifiers were controlled through stratification of age and duration of the procedure by employing the Chi-square test and p-value ≤ 0.05 was taken as statistically significant.

## Results

**Figure 1:**
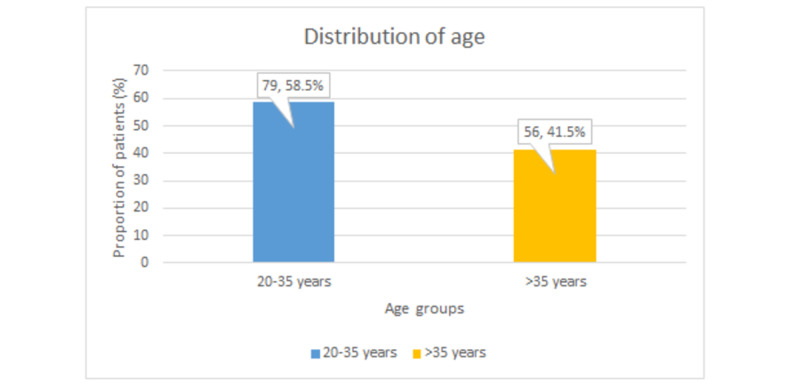
Age distribution of the participants The values depict (n,percentage %)

In a total of 135 participants, the mean age was 32.8±6.02 years. Nearly three-fifths (n=79 out of 135, 58.5%) of our participants were aged 20-35 years while others (n=56 out of 135, 41.5%) were aged >35 years, as shown in Figure 1.

**Figure 2:**
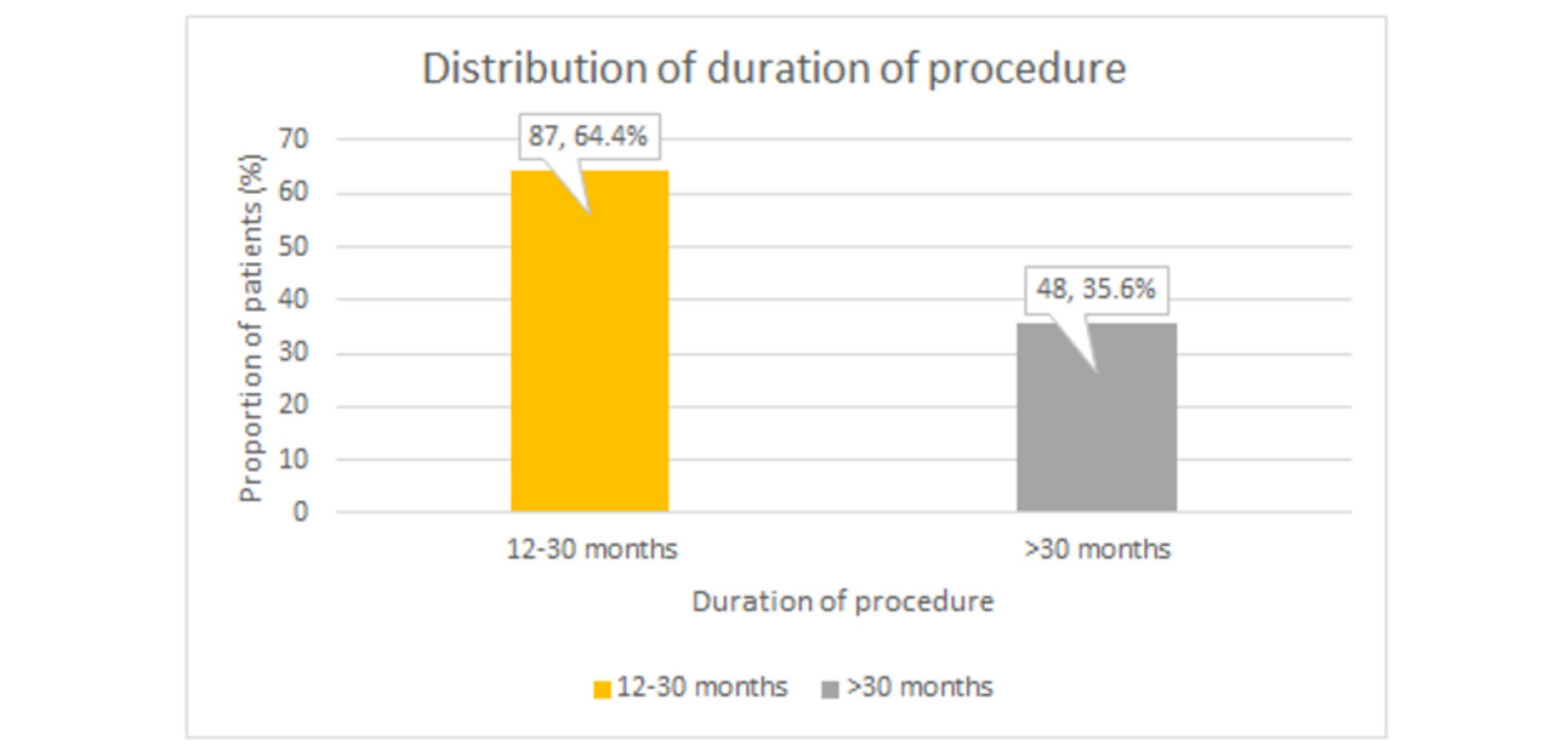
Distribution of participants with duration of procedure The values depict (n,percentage %)

The mean duration of the procedure with extravasation of our patients was 26.3±7.14 months. Nearly two-thirds (n=87 out of 135, 64.4%) of our participants had a mean duration of 12-30 months while other (n=48 out of 135, 35.6%) patients had a mean duration of >30 months, as represented in Figure 2.

**Figure 3:**
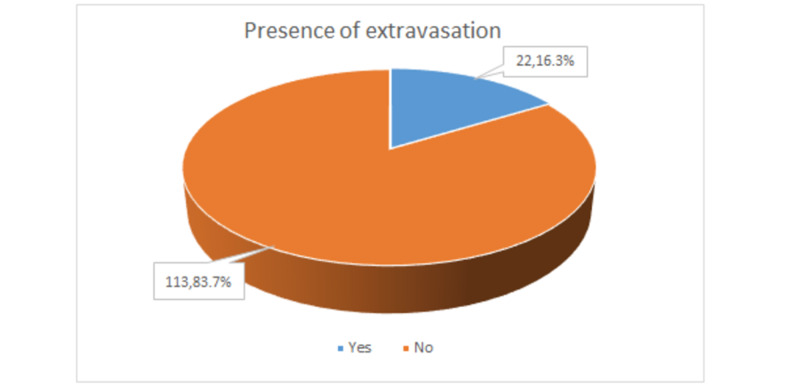
Frequency of extravasation cases The values depict (n,percentage %)

The extravasation cases were noted in less than one-fifth (n=22 out of 135, 16.3%) of patients while it was not observed in the rest of the participants (n=113 out of 135, 83.7%), as shown in Figure 3.

There was no significant association between age and presence of extravasation (p=0.315). Similarly, duration of the procedure (p=0.375) was also not significantly associated with presence of extravasation in this study. The frequency of extravasation was observed to be higher (n=15 out of 22, 68.2%) in participants aged 20-35 years. Nearly three-quarters (n=16 out of 22, 72.7%) of participants with extravasation had a procedure duration of 12-30 months, as shown in Table 1.

**Table 1:** Association of age and duration of procedure with extravasation

Variables		Presence of extravasation		p-value
		Yes (n=22 out of 135; 16.3%)	No (n=113 out of 135, 83.7%)	
Age	20-35 years	15 (68.2%)	64 (56.6%)	0.315
	>35 years	7 (31.8%)	49 (43.4%)	
Duration of procedure	12-30 months	16 (72.7%)	71 (62.8%)	0.375
	>30 months	6 (27.3%)	42 (37.2%)	

## Discussion

End to end anastomotic urethroplasty is the recommended treatment procedure for short-segment urethral strictures [<2 cm] but some controversy exists with time for the removal of the periurethral catheter during the post-operative period [4, 6]. Removal of the catheter has been performed from 10^th^ to 21^st^ postoperative day varying with the type of stricture repair, between 10^th^ and 14^th^ postoperative day in an anastomotic, and 21 or more post-operative days for graft repair [5, 12]. A study conducted by Hosam and his colleagues reported the extravasation rate to be 17% with catheter removal performed earlier at 3^rd^ postoperative day and assessment of leakage via post-operative MCU in patients with late removal of urethral catheter [5].

The post-operative removal of the urethral catheter is usually performed three weeks after surgery followed by the voiding cystourethrogram through the suprapubic catheter (SPC) tube to determine the integrity of the repair. In the case of satisfactory results, removal of SPC is performed one to three days later. A retrograde pericatheter urethrography (RPU) was performed before the removal of urethral catheters in our study. The urethral catheter was removed in cases demonstrating a normal urethral outline. Furthermore, in case of the presence of an SPC, the urethral catheter was clamped and removed after one to three days. It was retained for the addition of one to three weeks in cases in which contrast extravasation was observed based on the extent of extravasation.

This technique has multiple advantages including the depiction of the integrity of the urethral mucosa at the anastomotic site and the confirmation of urethral patency following urethroplasty. It enables us to identify patients in which retaining the catheter for a longer time (cases showing dye extravasation) may be beneficial. It helps to avoid unnecessary urethral manipulations such as removal and reinsertion of a catheter, which may damage the anastomosis or the patched area. This problem is often encountered in cases when voiding cystourethrogram is performed to confirm the integrity of urethral healing.

RPU had a success rate of 100% in our study and no complications as a result of the procedure were observed. Santucci and his colleagues in their study observed an extravasation rate of 1% in 168 patients in which anastomotic urethroplasty was performed for bulbar stricture with a post-operative catheterization for 14 days [13]. RPU showed contrast extravasation in 22 out of 135 (16.3%) of the subjects in our study. Prolonged catheterization was beneficial in these patients and satisfactory voiding was reported at the completion of the follow-up period. It has been published that pcRUG is the most beneficial radiologic diagnostic technique to evaluate the timing for urethral catheter removal following urethroplasty without the risk of re-catheterization [6, 7]. We observed similar results in our study that pcRUG is a reliable technique for urethral anastomotic healing assessment without the requirement of catheter removal. Etiology was reported to play a prominent role in the healing process in this study.

The findings of our study are comparable with multiple studies conducted in previous literature. In our study, the mean age of patients was found to be 32.8±6.02 years. In a study conducted by Bansal A, et al. the mean age of patients was 30.8 years while in another study by Solanki S, et al. the mean age of the patients was 32±13 years [8, 14]. In the present study, the mean duration of the procedure was noted as 26.3±7.14 months. The study of Bansal A, et al. also reported the duration of the symptoms at presentation to be three to seven months (mean 4.8 months) [14]. In this study, the prevalence of extravasation cases was found in 22 (16.3%) patients. Bansal A, et al. found the prevalence of contrast extravasation in 51 (14.8%) patients [14]. The study by Solanki S, et al. was conducted on two groups in which extravasation of dye was noted in four patients (33%) of the iatrogenic group and 14 patients (87.5%) of the post-traumatic group [8].

This study has a few limitations. Firstly, it was conducted at a single institution and the sample population was small. However, we believe our study provides a platform for further investigations to be conducted at multiple primary care settings on a larger sample size involving a variety of ethnic populations.

## Conclusions

Extravasation is prevalent in posterior urethral stricture patients. This offers essential clinical implications for the physicians to investigate as appropriate treatment and diagnosis are necessary to decrease the risk of postoperative complications in these patients.

## References

[1] Santucci RA, Joyce GF, Wise M (2007). Male urethral stricture disease. J. Urol.

[2] Palminteri E, Berdondini E, Verze P, De Nunzio C, Vitarelli A, Carmignani L (2013). Contemporary urethral stricture characteristics in the developed world. Urol.

[3] Hampson LA, McAninch JW, Breyer BN (2014). Male urethral strictures and their management.. Nat Rev Urol.

[4] Poelaert F, Oosterlinck W, Spinoit AF, Lumen N (2017). Duration of urethral catheterization after urethroplasty: how long is enough?. Minerva Urol Nefrol.

[5] Al-Qudah HS, Cavalcanti AG, Santucci RA (2005). Early catheter removal after anterioranastomotic (3 days) and ventral buccal mucosal onlay (7 days) urethroplasty. Int. Braz J Urol.

[6] Lee SC, Park SS, Choi HS (1995). The significance on the retrograde pericatheter urethrography in the timing of the removal of indwelling urethral catheter. Korean J Urol.

[7] Balogun BO, lkuerowo SO, Akintomide TE, Esho JO (2009). Retrograde pericatheter urethrogram for the post- operative evaluation of the urethra. Afr J Med Sci.

[8] Solanki S, Hussain S, Sharma DB, Solanki FS, Sharma D (2014). Evaluation of healing at urethral anastomotic site by pericatheter retrograde urethrogram in patients with urethral stricture. Urol Ann.

[9] Granieri MA, Webster GD, Peterson AC (2016). A critical evaluation of the utility of imaging after urethroplasty for bulbar urethral stricture disease. Urol.

[10] Sussman RD, Hill FC, Koch GE, Patel V, Venkatesan K (2017). Novel pericatheter retrograde urethrogram technique is a viable method for postoperative urethroplasty imaging. Int Urol Nephrol.

[11] Granieri MA, Webster GD, Peterson AC (2015). 1066 Extravasation on postoperative peri-catheter retrograde urethrogram after bulbar urethroplasty: time to pull the RUG out?. Eur Urol Suppl.

[12] Brian JF, Webstar GD (2004). Urethral stricture and disruption. Glen Urologic Surgery 6th edition.

[13] Santucci RA, Mario LA, McAninch JW (2002). Anastomotic urethroplasty for bulbar urethral stricture: analysis of 168 patients. J Urol.

[14] Bansal A, Singh V, Sinha R (2017). Retrograde pericatheter urethrography (RPU) technique and its clinical use after urethroplasty: a single center experience. Afr J Urol.

